# The Reliability of Contralateral Suppression of Otoacoustic Emissions Is Greater in Women than in Men

**DOI:** 10.3390/audiolres12010008

**Published:** 2022-01-19

**Authors:** W. Wiktor Jedrzejczak, Edyta Pilka, Malgorzata Pastucha, Krzysztof Kochanek, Henryk Skarzynski

**Affiliations:** 1 Institute of Physiology and Pathology of Hearing, ul. Mochnackiego 10, 02-042 Warsaw, Poland; e.pilka@ifps.org.pl (E.P.); m.pastucha@ifps.org.pl (M.P.); k.kochanek@ifps.org.pl (K.K.); skarzynski.henryk@ifps.org.pl (H.S.); 2 World Hearing Center, ul. Mokra 17, Kajetany, 05-830 Warsaw, Poland

**Keywords:** efferent, medial olivocochlear reflex, otoacoustic emissions, TEOAE, SOAE, reliability

## Abstract

The aim of this study was to compare the reliability of the medial olivocochlear reflex (MOCR) between men and women. The strength of the MOCR was measured in terms of the suppression of transiently evoked otoacoustic emissions (TEOAEs) by contralateral acoustic stimulation (CAS). The difference between TEOAEs with and without CAS (white noise) was calculated as raw decibel TEOAE suppression as well as normalized TEOAE suppression expressed in percent. In each subject, sets of measurements were performed twice. Reliability was evaluated by calculating the intraclass correlation coefficient, the standard error of measurement, and the minimum detectable change (MDC). The study included 40 normally hearing subjects (20 men; 20 women). The estimates of MOCR for both genders were similar. Nevertheless, the reliability of the MOCR was poorer in men, with an MDC around twice that of women. This can be only partially attributed to slightly lower signal-to-noise ratios (SNRs) in men, since we used strict procedures calling for high SNRs (around 20 dB on average). Furthermore, even when we compared subgroups with similar SNRs, there was still lower MOCR reliability in men.

## 1. Introduction

The effect of the medial olivocochlear reflex (MOCR) is to reduce the sensitivity of one cochlea (although sometimes it is seen to increase) when the contralateral cochlea is stimulated (reviewed in [1]). It is usually assessed in humans by measuring the reduction (in dB) of otoacoustic emissions (OAEs) when contralateral acoustic stimulation (CAS) is applied [2]. The measurement of the MOCR is of interest to researchers because it can simultaneously test peripheral and central processes related to hearing. There have been some intriguing findings: it has been shown that the MOCR is reduced when subjects are exposed to noise [3,4]; it can be used to track the development of frequency discrimination in noise [5], and to assess the ability to recognize speech in noise [6].

However, there are also many questions. Some studies report that attention has an effect on the MOCR [7,8], while others see no effect [9,10]. A recent review paper [11] concluded that the MOCR is unlikely to play a role in listening difficulties experienced by some children, even though previous reports have shown otherwise. A major limitation of several MOCR studies, especially some older ones, is that they are based on less-than-ideal experimental conditions, with the main problem being a generally poor signal-to-noise ratio (SNR).

The motivation for the present study was to take a step back from investigating the fine details and take a look at some basic MOCR characteristics, such as the effect of gender. If the MOCR is intended to detect pathology, or monitor performance on a test, it is necessary to know what change can be considered significant and what factors influence it. It is already known that Transiently evoked otoacoustic emissions (OAEs) have a higher average magnitude in females than in males (e.g., [12]). Likewise, we know that the prevalence of spontaneous OAEs (SOAEs) is higher in females than in males, and ears with SOAEs have generally larger evoked OAEs as well [13]. Since MOCR effects are relatively small, it is quite possible that any gender effects will affect MOCR effects. It therefore seems important to investigate whether gender and SOAE presence have an effect on the MOCR, and a key parameter here is reliability, a measure that has not yet been adequately investigated. There have been a handful of studies in this area, but the information so far is incomplete. Stuart and Kerls (2018) [14] reported that there was no laterality or gender effect in terms of raw decibel change to the MOCR, but they did see a gender effect in terms of percentage change, with men having a slightly higher effect. Some recent studies have also shown that the presence of SOAEs affects MOCRs when they are measured using evoked OAEs [15,16,17]. It has also been shown that the MOCR effect appears larger when using SOAEs rather than evoked OAEs, and that the presence of SOAEs increases MOCR detectability when evoked OAEs are studied [18,19]. From this work, one can conclude that the presence of SOAEs seems to be a relatively important factor when measuring MOCRs.

Importantly, there seems to be little information on whether there are gender differences in MOCR reliability. Some studies have evaluated groups consisting of men and women combined [20,21,22,23,24,25], while others have considered only women [17,26]. Of all these studies, only Stuart and Cobb [21] investigated the effect of gender. They did not find any effect of gender on MOCR reliability, although they studied only a relatively small group and did not check for the presence of SOAEs.

In summary, the purpose of the present study was to assess whether there were any differences in the reliability of the MOCR between men and women. A secondary goal was to determine whether the presence of SOAEs affected MOCR reliability, in either men or women. In seeking to answer these questions, our approach was to use the highest possible SNRs.

## 2. Materials and Methods

### 2.1. Participants

Measurements were performed in a group of 40 normally hearing adults (20 women, average age 27.8, standard deviation (SD) = 7.7 years; 20 men, average age 28.7, SD = 7.5 years). All subjects underwent visual inspection of the ear canal and tympanic membrane of both ears, followed by tympanometry, acoustic reflex threshold (ART) measurement, pure tone audiometry, and OAE measurement.

Pure tone audiometric testing was conducted using the Madsen Astera (GN Otometrics). All subjects had pure tone thresholds for air conduction better than 25 dB HL between 0.5 and 8 kHz. Middle ear function was examined using the Titan device (Interacoustics, Middelfart, Denmark). Normal middle ear function was verified using 226 Hz tympanometry (peak pressure between –100 and +100 daPa and peak-compensated static acoustic admittance of 0.2–1.0 mmhos) and ipsilateral and contralateral ARTs (for clicks and 0.5–4 kHz tones). In all subjects, ARTs were above 80 dB SPL, i.e., well above the levels used for the OAE suppression measurements described below. All subjects had no known history of otologic disease.

### 2.2. Procedures

Transiently evoked otoacoustic emissions (TEOAEs) were measured using an ILO 292-II system, software version ILOv6 (Otodynamics Ltd., Hatfield, UK). Measurements were made in a sound booth. In each subject, measurement of the effect of CAS on TEOAEs was done twice without refitting the probe. The assumption here was that measurements made without refitting provide the best possible reliability estimates. This approach also eliminates other sources that can influence test–retest reliability (such as accuracy of probe fitting), and so remaining differences relate mostly to inherent fluctuations of the signal and noise under the paradigm used. Measurements were made in a single session with a break of about 15 s between each set. Before a session, the probes were calibrated using the cavity provided by the manufacturer.

The standard ILO protocol for measuring contralateral suppression of TEOAEs was used: 65 dB peSPL clicks (linear mode) were delivered to one ear and 60 dB SPL noise to the contralateral ear (2 s on/off time). Clicks were delivered at a rate of 50 per second, giving an acquisition window of 20 ms. To minimize stimulus artifacts, the initial part of the response (0–2.5 ms) was automatically windowed out by the system. Each of the two measurements used 1000 averages (the maximum for this system), compared to the standard 260 used in most studies with this equipment. Note that the ILO system counts 1 measurement as a sequence of four stimuli, and there are two response buffers, so 1000 averages means that 8000 clicks were used for each condition (with and without CAS). The requirement was that all recordings should have an SNR of at least 9 dB for recordings with and without CAS. Signal parameters were analyzed as global values. Only right ears were tested (TEOAEs were measured in the right ear and CAS was delivered to the left). Responses were automatically filtered by the system over 400–6400 Hz (this response represented the global value). A default artifact rejection level of 6 mPa was used. Measures of TEOAE response level, SNR, and TEOAE suppression were used for analysis. The study focused on global MOCR as it has been shown that half-octave band values have significantly lower reliability and therefore reduced practical utility [16]. SNR was calculated by subtracting the noise level (in dB) from the response level (in dB). MOCR was calculated by two methods. First, the response level with CAS was subtracted from the level without—the raw decibel measure. The second method took account of phase effects and was based on the percentage change in the time domain waveforms [27,28]:

(1) $$\Delta_{MOC}=100\times \sqrt{\frac{1}{N}\sum_{n=1}^{N}{\left(a_{quiet}\left[n\right]-a_{noise}\left[n\right]\right)}^{2}}\;/\sqrt{\frac{1}{N}\sum_{n=1}^{N}{\left(a_{quiet}\left[n\right]\right)}^{2}\;},$$

where *N* is the number of samples, *a_quiet_* is the amplitude of the TEOAE waveform measured without CAS, and *a_noise_* is the amplitude of the TEOAE waveform measured with CAS. It was shown that MOCR affects TEOAEs in two ways: by reducing magnitude and by reducing latency (e.g., [29]). Therefore, it might be expected that MOCR calculated by the second method would show the greater effect.

SOAEs were acquired using the in-built routine provided by the ILO 292 equipment, resulting in a recording of so-called synchronized SOAEs (SSOAEs). To do this, OAEs evoked by click stimuli of 80 dB SPL were recorded in an 80 ms window (click rate of 12 s^−1^) and the first 20 ms of each averaged response (containing largely the evoked part) was discarded. The spectra of responses from the remaining 60 ms were analyzed in search for SSOAEs. An ear was classified as “with SSOAEs” when at least one peak was found in the spectrum that exceeded the noise floor by 6 dB. For each gender, there were 10 ears with SSOAEs (SSOAE+) and 10 ears without SSOAEs (SSOAE−).

The dataset was constructed in a way to have equal numbers by gender and SSOAE presence. The number does not reflect the prevalence of SSOAEs by gender, which is higher in women.

To prevent subjects from falling asleep during the tests, which could cause artifacts (e.g., snoring, change of position), a movie was shown with the sound track muted. As shown by recent studies, such an experimental design does not seem to influence the major TEOAE parameters or suppression levels of TEOAEs by CAS [9,10].

### 2.3. Data Analysis

All analyses were made in Matlab (version 2018b, MathWorks, Natick, MA, USA). For all measured parameters, the statistical significance of mean differences was evaluated using repeated-measures analysis of variance (rmANOVA). Post hoc tests were conducted using a *t*-test when the data fulfilled a criterion of normality, otherwise a Wilcoxon rank-sum test was used.

The reliability of measurements was evaluated by the two-way random effects intraclass correlation coefficient (ICC) and the standard error of measurement (SEM). SEM was calculated as $\mathrm{STD}\cdot \sqrt{1-\mathrm{ICC}}$, where STD is the standard deviation of the combined test and retest MOCR estimate. These measures have been used in previous studies of MOCR reliability [16,17,30]. When the SEM value grows, the reliability is poorer. Based on the SEM, the minimum detectable change (MDC) can be determined; if the confidence interval is set at 95%, then the MDC is given as $\pm 1.96\cdot \sqrt{2}\cdot \mathrm{SEM}$.

## 3. Results

Although our main focus was MOCR analysis, we start by showing a basic characteristic of TEOAE parameters—that there are distinct differences between gender groups. An rmANOVA was used to examine differences in global values of TEOAE response levels and SNRs as a function of gender, SSOAE presence, and test (first and second measurement). For global response levels, it was found that there was a significant effect of gender (F(1,36) = 15.9, *p* < 0.001), SSOAE presence (F(1,36) = 65.7, *p* < 0.001), and no effect of test or any interactions. For global SNR, there was a similar situation, with significant effects being gender (F(1,36) = 16.5, *p* < 0.001) and SSOAE presence (F(1,36) = 56.1, *p* < 0.001).

Average global response levels and SNRs of TEOAEs of the studied groups are shown in Table 1. It can be seen that response levels and SNRs are higher for women by around 3.5 dB. They are also higher for ears with SSOAEs by at least 5 dB.

These results show clear differences between TEOAEs of men and women, and ears with and without SSOAEs. The next step was to test whether these gender and SSOAE differences also had an effect on the MOCR, in particular its reliability.

An rmANOVA was used to examine the differences in MOCR (for both measures—raw dB and %) as a function of gender, SSOAE presence, and test. Unlike response levels and SNRs, there was no significant main or interaction effect.

Table 2 shows average MOCR expressed as raw decibel and total effect expressed as percentage. The raw MOCR effect was on average 0.7 dB for both genders, while the total effect was 23% in men and 21% in women. In women, the MOCR estimates in ears with SSOAEs were higher than in ears without SSOAEs (however, rmANOVA showed no significant SSOAE effect). Interestingly, in men, the MOCR estimates were very similar between ears with and without SSOAEs.

Turning to the main focus of this study, i.e., the MOCR reliability for men and women, Table 3 shows ICC, SEM, and MDC of the MOCR (expressed as raw dB and %). Note that the SEM is higher for men. In terms of raw dB, it is around two times higher for all subjects as well as for ears with and without SSOAEs. However, the situation is different for total percentage MOCR. For women, the SEM is similar for all subjects and for ears with and without SSOAEs; for men, the SEM is lower for ears with SSOAEs but higher for ears without SSOAEs. For ears with SSOAEs, the SEM for men is about the same as for women (1.34 compared to 1.11, respectively), while it is much higher for ears without SSOAEs (2.47 compared to 0.97, respectively).

## 4. Discussion

This study has shown that the MOCR in men has poorer reliability than in women. It also appears that MOCR reliability is not directly related to the presence of SSOAEs. Indeed, it seems that the lower MOCR reliability in men is not entirely due to SNR, but is an intrinsic characteristic of gender.

The values of raw MOCR, as measured in decibels, are similar to those in previous studies on the same equipment (e.g., [14,17,24]). The present study also confirms the results of Stuart and Kerls [14] regarding the lack of difference in MOCR between men and women in terms of raw decibel. However, Stuart and Kerls [14] also showed that, in terms of percentage, the MOCR was significantly higher in men than in women. Here, there was a slightly higher value for men, although rmANOVA showed no clear effect of gender. The lack of significance may be due to the smaller size of the group here (20, c.f. 50 in [14]).

The data were collected such as to have groups with equal numbers of subjects. Thus, they do not reflect the prevalence of SSOAEs, which is higher in women [13]. While we did see a significant effect of SSOAE presence on response level and SNR, in this work the difference in MOCR between ears with and without SSOAEs was not significant. This may simply reflect the smaller size of the dataset. It was similarly not present in an earlier study of small sample size [20], and was only discovered after larger datasets were employed [15,17]. For women, the results were similar to those of [17], with the MOCR being higher for ears with SSOAEs. However, the difference was not statistically significant, and is most likely related to spread of the data and a smaller number of ears. Curiously, for men, the MOCR strength was nearly identical for ears with and without SSOAEs. It could be that the effect of SSOAEs on the MOCR is present only for females, and this needs to be confirmed in a study with a larger group of subjects.

The main new result from this study is that men have less reliable MOCR measures than do women. The difference is quite marked, expressed numerically as around two times higher SEM. In a clinical setting, the difference could be meaningful, with the MDC also twice as large. The decrease in reliability can be partially attributed to a slightly lower SNR in men, but not completely. If two subgroups are compared, one in which the SNR is slightly higher in men than in women (i.e., men with SSOAEs who have an average SNR of 21.3 dB and women without SSOAEs who have an average SNR of 17.7 dB), the MDC for MOCR in terms of raw decibel is still more than twice as large for men as for women (0.49 vs. 0.21 dB). This suggests that SNR may not be the only issue involved but that it could be an inherent gender characteristic. As another point of comparison between the genders, in women, the reliability of both MOCR measures is similar for ears with and without SSOAEs, while for men, the total MOCR reliability was lower for ears without SSOAEs, which had nearly a two times higher SEM.

It should be underlined that the present study focused on global MOCR, i.e., for the whole length of the signal and over its entire frequency range. Time–frequency analysis of TEOAEs indicates that often multiple reflection components can be distinguished [31,32,33]. It would seem logical that estimating MOCR based on a strict time–frequency range, or even analysis of particular components, might provide an advantage. In addition, some studies have used different windows or frequency ranges for MOCR estimates [34]. However, our previous studies on MOCR have clearly shown that reliability decreases when shorter windows are used (i.e., a selected time range for the TEOAE waveform) and similarly when half-octave band frequency ranges are used instead of global values [16,17,30]. Furthermore, even more advanced time–frequency processing (e.g., use of matching pursuit) does not seem to provide any advantage [10].

Finally, it should be highlighted that the strength of the above results is supported by relatively high SNR criteria. Many MOCR studies are based on data collected with quite low SNRs, e.g., 3 dB or sometimes 6 dB (the latter being only for the CAS− condition, with the CAS+ condition then possibly having an SNR below 6 dB). Here, best reliability — the lowest SEM and MDC — were for women with SSOAEs. They also had the highest SNRs (on average 26 dB) giving an MDC of 0.21 and an average raw effect of 0.9 dB. For the other subgroups, higher MDCs were obtained for lower MOCR effects. In the face of this evidence, it seems that a criterion of 6 dB, which applies to standard OAE tests, might not be sufficient for MOCR testing, and this certainly seems to be the case for a 3 dB criterion. The findings of all such studies should be treated with considerable caution. Another conclusion flowing from our findings is that for basic experiments where small MOCR differences are of interest, female subjects who have SSOAEs are a natural preference.

It is worth noting some limitations of the current study. Of course, the groups could be enlarged, but they are nevertheless bigger than those in most previous MOCR studies that have measured reliability (e.g., [20,21,22,23,24,25]). McMillan and Hanson [35] suggest that at least 40 subjects should be used for reliability studies. Although the subgroups used here were smaller, the results are still comparable to those of previous studies using larger groups [14,17].

In the case of MOCR measurements based on CAS, it is always possible for the signal to be contaminated by middle ear muscle responses (MEMRs), e.g., [36]. We did not use any special procedure to check for MEMRs within our TEOAE measurements, as our equipment did not provide such a facility. Nevertheless, ARTs in this work were at least 80 dB, which is well above the 60 dB of noise used for CAS. Some related studies point out that at levels of about 60 dB, only a fraction of ears exhibit MEMRs [37].

Of course, it would also be nice to use even higher SNRs. However, we did use the highest possible number of averages the system offers, requiring around 10 min for a single TEOAE CAS+/CAS− recording. We think it would be practically impossible to get higher SNRs with this equipment as some subjects would find the longer test times difficult.

Finally, it should be mentioned that it is hard (and maybe even impossible) to definitively separate effects of gender, SSOAEs, and SNR on MOCR and its reliability. Thus, it can only be hypothesized what is cause and what is effect here. Nevertheless, our results might inspire experiments on larger or possibly better selected groups. Additionally, it seems crucial to develop methods of fast MOCR measurements that at the same time provide high SNR.

## 5. Conclusions

The present study showed that MOCR reliability is lower in men than in women. This can be partially attributed to lower SNRs in men, but it also seems to be a gender characteristic. This is clearly seen in the case of ears that do not have SSOAEs, where men in general had lower reliability.

## Figures and Tables

**Table 1:** Average global response levels and SNRs of TEOAEs of studied groups of men and women together with standard deviations (in brackets). Additionally, *p*-values for comparisons between subgroups of men and women are shown. The data are divided according to SSOAE presence. Key: *N*, number of subjects/ears in each subgroup; SSOAE+, ears with SSOAEs; SSOAE−, ears without SSOAEs.

	*N*		Response Level (dB SPL)			SNR (dB)		
Group	Men	Women	Men	Women	*p*-Value	Men	Women	*p*-Value
All	20	20	9.3 (4.9)	13.0 (4.9)	0.021	18.3 (4.5)	21.9 (5.1)	0.031
SSOAE+	10	10	12.9 (3.3)	16.9 (3.5)	0.018	21.3 (4.1)	26.0 (3.3)	0.017
SSOAE−	10	10	5.6 (3.0)	9.1 (2.2)	0.0079	15.2 (2.3)	17.7 (2.2)	0.023

**Table 2:** Average MOCR shown as raw effect (in dB) and total effect (in %) (standard deviations in brackets). The data are divided according to SSOAE presence. Key: *N*, number of subjects/ears in each subgroup; SSOAE+, ears with SSOAEs; SSOAE−, ears without SSOAEs.

	*N*		MOCR Raw (dB)		MOCR Total (%)	
Group	Men	Women	Men	Women	Men	Women
All	20	20	0.7 (0.4)	0.7 (0.5)	23.0 (10.0)	21.4 (7.8)
SSOAE+	10	10	0.8 (0.5)	0.9 (0.6)	22.6 (14.1)	25.5 (8.5)
SSOAE−	10	10	0.7 (0.4)	0.5 (0.4)	23.3 (3.3)	17.4 (4.3)

**Table 3:** ICC, SEM, and MDC of MOCR for raw effect (in dB) and total effect (in %). The data are divided according to SSOAE presence. Key: ICC, intraclass correlation coefficient; SEM, standard error of measurement; MDC, minimum detectable change for 95% interval; *N*, number of subjects/ears in each subgroup; SSOAE+, ears with SSOAEs; SSOAE−, ears without SSOAEs.

		MOCR Raw (dB)		MOCR Total (%)	
Group	Measure	Men	Women	Men	Women
All	ICC	0.83	0.97	0.96	0.98
	SEM	0.17	0.08	2.00	1.05
	MDC	0.48	0.22	5.55	2.92
	*N*	20	20	20	20
SSOAE+	ICC	0.88	0.98	0.99	0.98
	SEM	0.18	0.08	1.34	1.11
	MDC	0.49	0.22	3.73	3.07
	*N*	10	10	10	10
SSOAE−	ICC	0.70	0.97	0.61	0.94
	SEM	0.16	0.08	2.47	0.97
	MDC	0.45	0.21	6.85	2.68
	*N*	10	10	10	10

## Data Availability

The data underlying this article will be shared on reasonable request to the corresponding author.
